# Solitary choroidal metastasis from gastric adenocarcinoma: a case report

**DOI:** 10.1186/s40792-017-0311-5

**Published:** 2017-02-21

**Authors:** Masanari Shimada, Susumu Amaya, Hiroaki Sugita, Yuichiro Furutani, Yoshinori Munemoto, Takeshi Mitsui, Toshiro Tanahashi, Yuzo Kikuchi

**Affiliations:** 1 Department of Surgery, Fukui-ken Saiseikai Hospital, 7-1 Wadanaka-Funabashi, Fukui, 918-8503 Japan; 2 Department of Ophthalmology, Fukui-ken Saiseikai Hospital, Fukui, 918-8503 Japan; 3 Department of Radiation therapy, Fukui-ken Saiseikai Hospital, Fukui, 918-8503 Japan

**Keywords:** Gastric cancer, Adenocarcinoma, Choroidal metastasis, Adjuvant chemotherapy, Solitary

## Abstract

**Background:**

Metastatic choroidal carcinomas that originated from the gastrointestinal tract are extremely rare. We report a case of suspected solitary choroidal metastasis from gastric adenocarcinoma.

**Case presentation:**

The patient was a 60-year-old man who had undergone laparoscopic distal gastrectomy with D1+ lymphadenecetomy for gastric cancer. The clinical stage was T1bN0M0 (TNM classification), but the pathological stage was T4aN0M0 beyond expectation. Adjuvant chemotherapy with oral Tegafur, Gimeracil, Oteracil potassium (TS-1^®^) was initiated. But he suddenly complained of decreased visual acuity in his right eye about 8 months later. This was suspected to be caused by choroidal metastasis of gastric adenocarcinoma. Chemotherapy with paclitaxel (PTX) and intensity-modulated radiotherapy (IMRT) achieved complete remission and spared the patient from going blind.

**Conclusions:**

This case demonstrates that we should be aware of the possibility of choroidal metastases, when visual symptoms arise during treatment of gastric cancer.

## Background

Choroidal metastatic tumors from gastrointestinal tract malignancies are extremely rare compared with more common metastatic breast and pulmonary carcinomas. The uvea consists of the iris, ciliary body, and choroid. In a study of 420 patients with uveal metastasis, 196 came from primary cancers of the breast (47%), 90 from the lung (21%), and 18 from the gastrointestinal tract (4%). Moreover, metastasis was found in the iris in 90 (9%), ciliary body in 22 (2%), and choroid in 838 (88%) of the 950 metastatic foci in the uvea [1]. Above all, choroidal metastases from gastric adenocarcinoma have been reported in only a few cases in the literature [2–4]. We report a rare case diagnosed as solitary right choroidal metastasis from gastric adenocarcinoma that was treated successfully.

## Case presentation

**Fig. 1:**
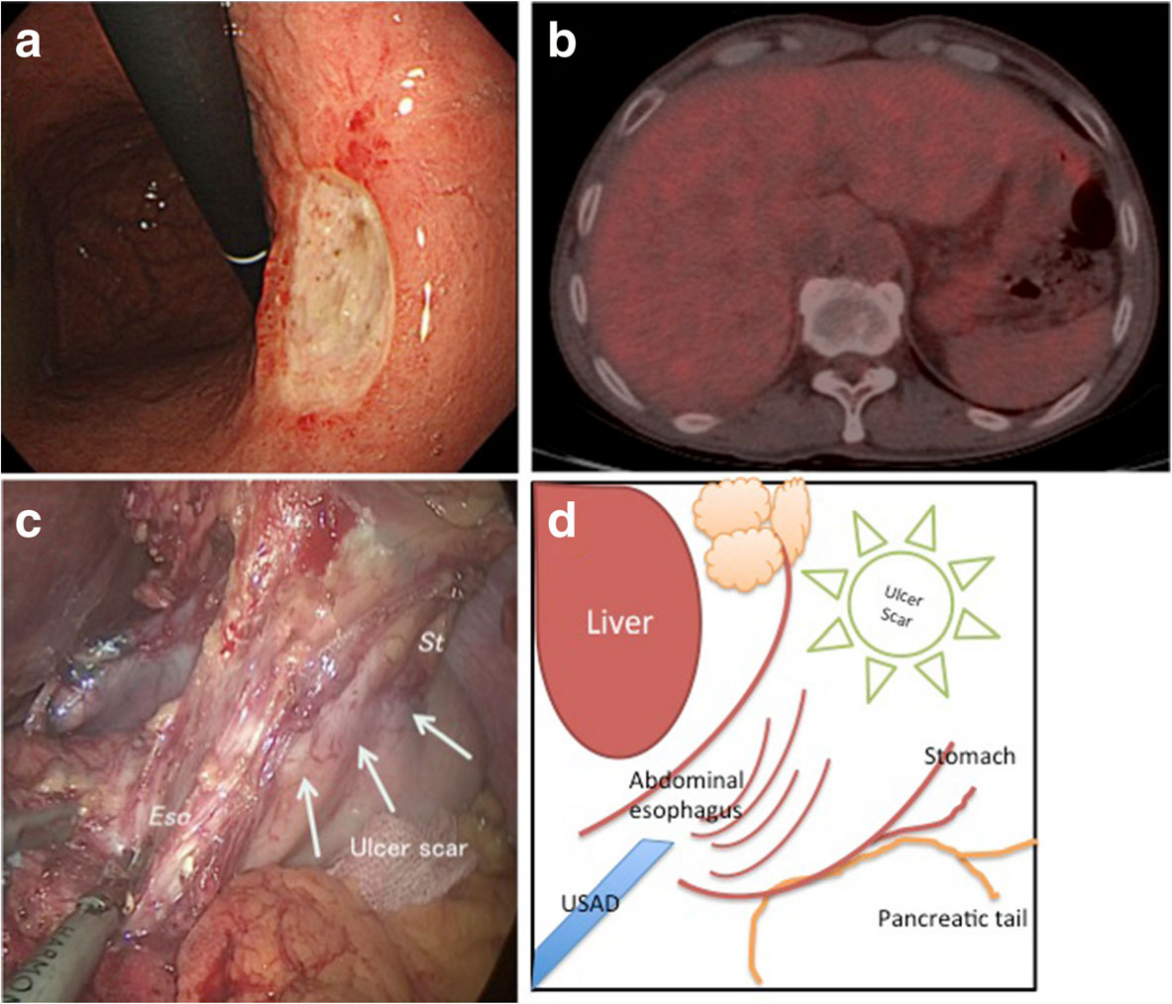
Preoperative examinations and intraoperative findings. **a** Esophagogastroduodenoscopy revealed an IIc+III lesion and a signet ring cell carcinoma on biopsy. **b** FDG-PET/CT could not detect other lymphnode or organ metastases and could not detect the exposure of the tumor at the serosal surface of the stomach. **c** The lesion was identified as an ulcer scar during laparoscopic distal gastrectomy. St: Stomach, Eso: esophagus. **d** Schema of Fig. 1C. USAD: ultrasonic-activated device

**Fig. 2:**
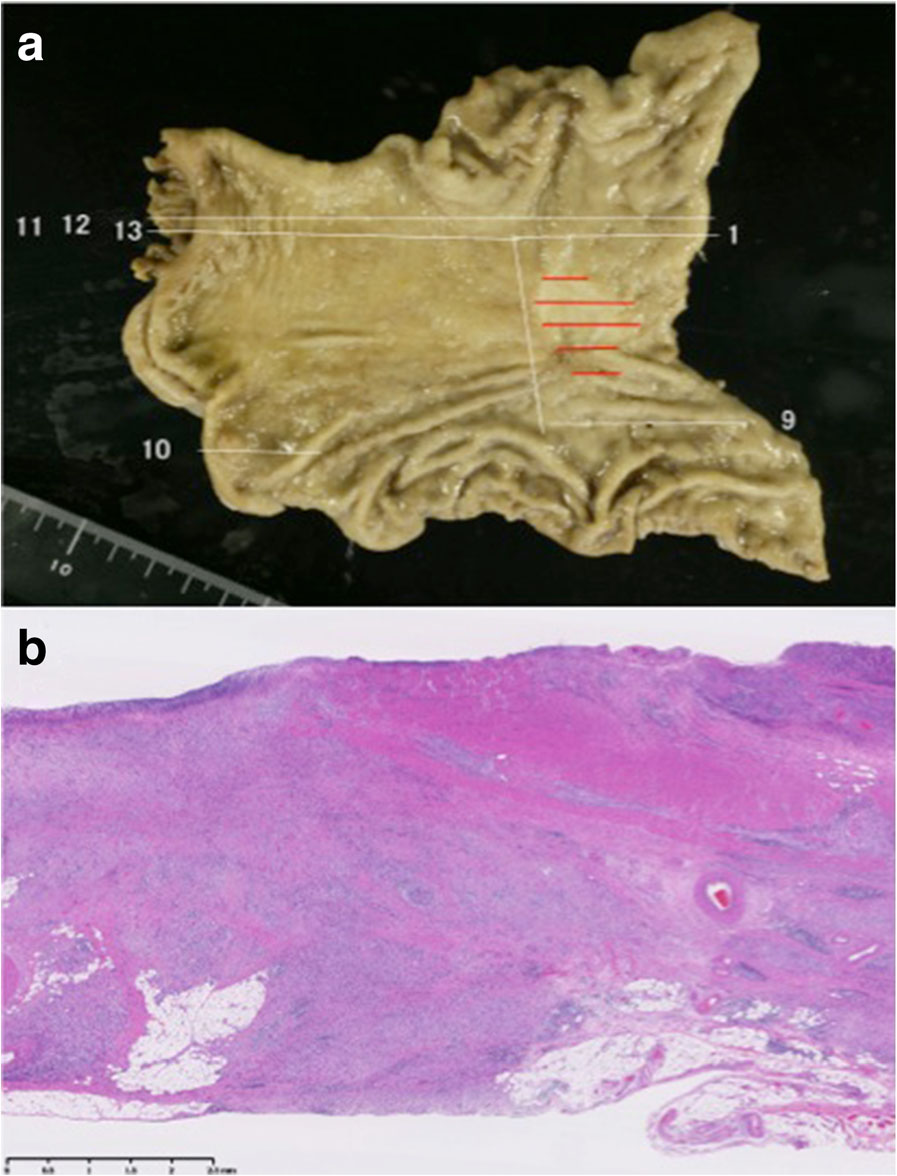
Pathological findings. **a** Resected specimen shows a cancer measuring 30 × 22 mm in the form of superficial depressed and excavated type. **b** Histopathological findings show poorly differentiated adenocarcinoma being exposed at the serosal surface only in the slice #5 only (hematoxylin and eosin stain, ×40)

**Fig. 3:**
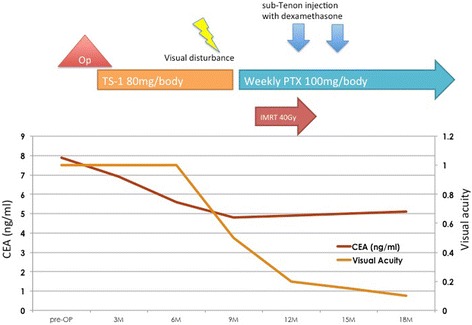
Clinical course. (M: months; Op: operation; TS-1: Tegafur, Gimeracil, Oteracil potassium; PTX: paclitaxel; IMRT: intensity-modulated radiotherapy; CEA: carcinoembryonic antigen)

A 60-year-old man was detected with an abnormality in his medical checkup. Esophagogastroduodenoscopy revealed an ulcerative lesion on the lesser curvature of the stomach (Fig. 1a). Gastric biopsy specimens from the ulcerative lesion showed signet ring cell carcinoma. We performed computed tomography (CT) and fluorodeoxyglucose-positron emission tomography (FDG-PET), but this did not reveal the primary lesion or lymphnode metastasis (Fig. 1b). We made the clinical diagnosis of T1bN0M0 gastric cancer in TNM classification and performed a laparoscopic distal gastrectomy with D1+ lymphadenectomy (Fig. 1c, d). Pathological findings showed exposure of the tumor at the serosal surface at only a single site in the specimen (Fig. 2a, b). We could not recognize the tumor penetration of the serosa intraoperatively. The finding of (tumor exposes on the surface of serosa) T4a was first detected microscopically. The final pathological diagnosis was MU, less, post, type 0-IIc+III (advanced), 30 × 23 mm, por2>sig, pT4a, sci, INFc, ly1, v1, pN0 (0/40), PM0 (8 mm), and DM0 (90 mm, HER2 score 0). We performed preoperative endoscopic marking by clips to 2-cm endoscopic margin and confirmed that the clips were existed in the resected specimen. But, the proximal resected margin was 8 mm in pathological findings, because the submucosal invasion was more widespread than the macroscopic margin. Thus, the pathological stage was T4aN0M0 beyond expectation. He began a year of adjuvant chemotherapy with Tegafur, Gimeracil, Oteracil potassium (TS-1^®^) 80 mg/body. However, he suddenly complained of visual disturbance in his right eye about 8 months later (Fig. 3: Clinical course).

**Fig. 4:**
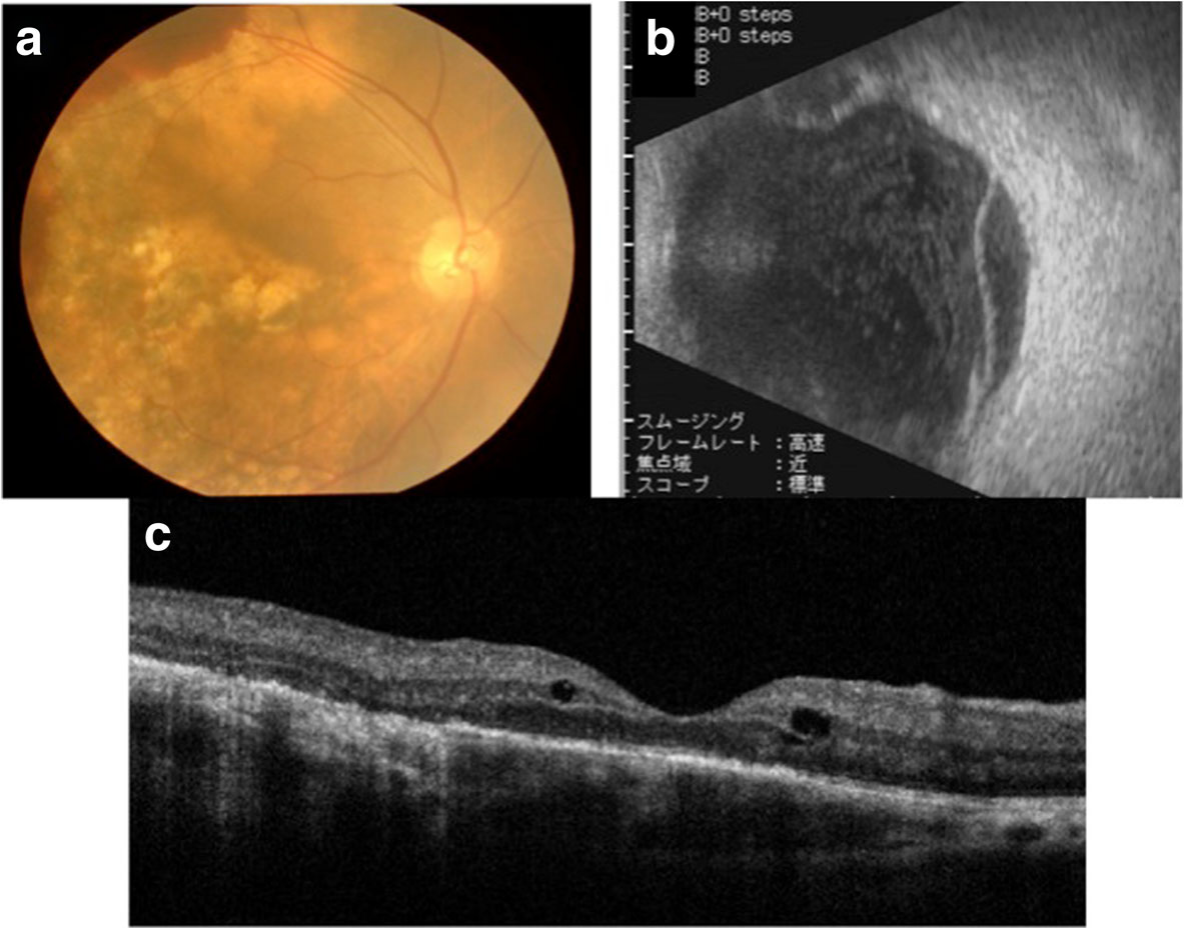
Ophthalmologic examinations. **a** Fundoscopic examination. **b** Ultrasonography. **c** Optical coherence tomographic examination

**Fig. 5:**
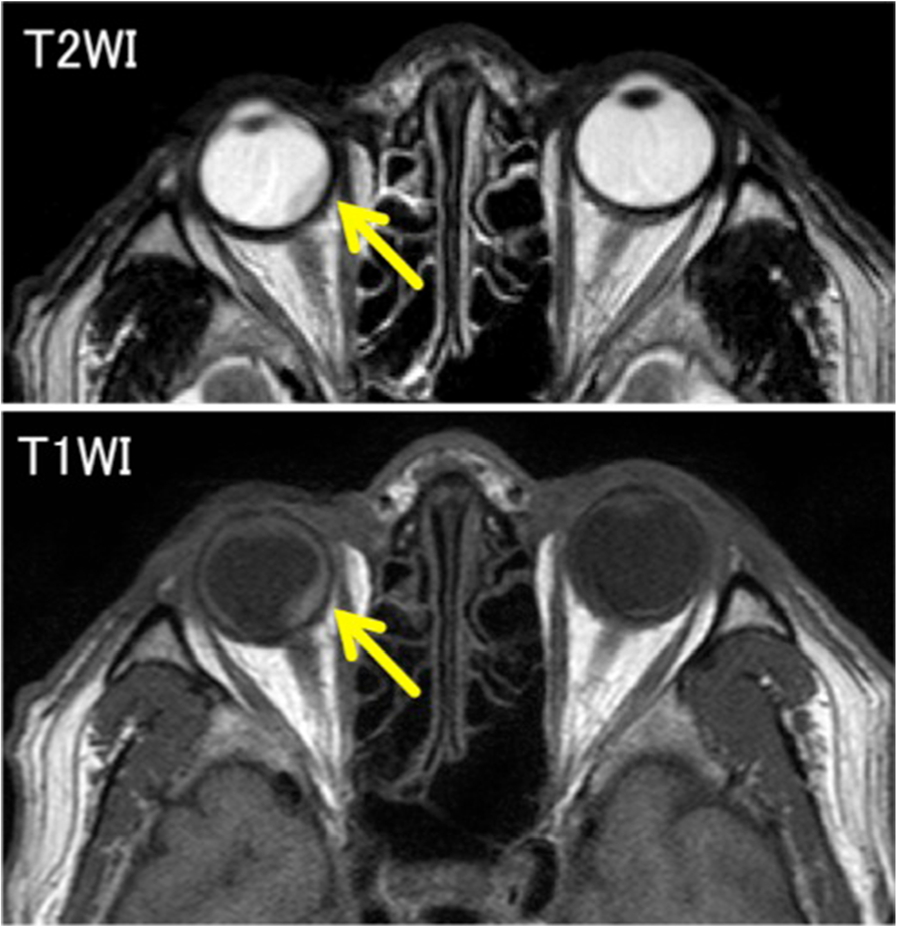
Magnetic resonance imaging (MRI). An elevated choroidal tumor was detected. The T1-weighted image was isointense, and the T2-weighted image was hypointense

To evaluate the cause of the visual disturbance, fundoscopic examination and complete ophthalmologic examinations were performed (Fig. 4). Fundoscopic examination revealed profuse exudate, subretinal hemorrhage, and retinal detachment suggesting metastatic choroidal tumor. Ultrasonography showed retinal detachment by choroidal tumor, and optical coherence tomographic examination showed macular edema and irregular elevation of choroidal tissue. Magnetic resonance imaging (MRI) was also performed and a smooth and sharply marginated tumor was detected in his right ocular fundus in T1- and T2-weighted images (Fig. 5). We could not detect any other origins for metastatic tumor of the choroid, although we performed CT, endoscopy, and FDG-PET.

We started the second line chemotherapy with weekly intravenous paclitaxel (100 mg/body) and intensity-modulated radiotherapy (IMRT) (40 Gy) to the right orbit. Although his retinal detachment was gradually worsened, sub-Tenon injection with dexamethasone was performed. Consequently, the retinal detachment was gradually improved and he was spared from going blind. There was a gradual decrease in carcinoembryonic antigen (CEA) from 7.9 ng/ml at diagnosis to 5.1 ng/ml after the chemoradiation therapy. He was continued to be treated with paclitaxel (PTX), 14 courses in total. At this point, we identified a complete response on PET and fundoscopic examination, and then we changed his chemotherapy to capecitabine (1200 mg/body) alone. His recurrence-free survival period is 3 years until now (Fig. 3).

### Discussion

It is considered that a number of reports about metastatic choroidal tumor have been increasing with the increase of cancer patients and improvement of survival rates with the advance of treatment. There are few reports about the cases from gastric adenocarcinoma [2–4]. Moreover, these reports just only show the choroidal metastases as one of the sites of systemic metastases, and there is no report about a solitary choroidal metastasis pointed out during adjuvant chemotherapy and achieved good local control.

Choroidal metastasis is considered to occur by hematogenous spread. This seems to be common in cases of lung cancer and breast cancer. In the English language literature, we could not find any previous articles about the risk or morbidity rate limited to only gastric cancer owing to its rarity. In our case, the microscopic vascular invasion of poorly differentiated adenocarcinoma or signet ring cell carcinoma was thought to be involved in the choroidal metastasis. Also, in approximately half of these patients, their primary sites are undetected despite the systematic evaluation, and they die of diffuse metastatic disease and their primary sites remain unknown [1].

In the diagnosis, it is important to differentiate between malignant melanoma and metastatic tumor, because melanoma and metastatic tumors are the two most common malignant choroidal tumors [5]. Malignant melanoma is seen as a localized lesion on fundoscopic examination in general; therefore, our findings were compatible with metastatic adenocarcinoma. In the literature, it has been reported that N-isopropyl-*p*-(^123^I) iodoamphetamine single-photon emission computed tomography (^123^I-IMP SPECT) and MRI are useful in distinguishing the two [6, 7]. We were appreciative of the accuracy of MRI, and the T1-weighted image was isointense and the T2-weighted image was hypointense, so their findings were compatible with metastatic tumor, and not melanoma. Although we could not provide a definitive diagnosis to our case because of the difficulty of choroidal biopsy, we diagnosed metastasis of gastric adenocarcinoma with the findings from various imaging studies. Because some authors have reported the diagnosis by fundoscopic findings alone in previous literatures [2, 4], we assume that direct biopsy may not be necessary for diagnosing clinically.

Chemotherapy and external beam radiotherapy may be effective. In our case, chemoradiation therapy (CRT) achieved complete remission and saved the patient from going blind as well. It is reported that choroidal metastasis from gastric adenocarcinoma is almost resistant to various therapies and is presented with multiple organ metastasis and severe prognosis [1, 3]. It is interesting that multimodal therapy was effective in our case and local control for the metastatic ocular lesion was achieved. Although the issue here is how we can explain the success in the treatment in this case, it is reasonable to assume that the metastatic choroidal lesion was fortunately localized within a small range and the symptom was detected in an early stage. The reason why we chose PTX is that we considered PTX as the second line. We thought standard TS-1 and cisplatin regimen will not be effective, because the choroidal metastasis was occurred during the adjuvant therapy with TS-1. Furthermore, we could not treat aggressively with a platinum-based combination regimen because of his chronic pancytopenia. On the other hand, we estimated that there is the risk of recurrence or other metastasis in several years, even once he had a complete remission. Although we debated whether or not to resume to treat with TS-1, TS-1 was predicted to have no effect in the first adjuvant chemotherapy. So, another oral anticancer drug that is approved for the management of gastric cancer was considered to be the best choice. Although there were few clinical trials for the efficacy of capecitabine for gastric cancer alone [8, 9], we considered capecitabine feasible for maintenance therapy after CRT. Currently, we intend to continue some kind of systematic chemotherapy for some time in the future with careful observation.

## Conclusions

We reported a rare case of gastric adenocarcinoma who presented with unilateral solitary choroidal metastasis during adjuvant chemotherapy. Therefore, it is important to be aware of the possibility of a metastatic choroidal tumor, when visual disturbances do arise during gastric cancer treatment.

## Abbreviations

^123^I-IMP SPECT: N-isopropyl-*p*-(^123^I) iodoamphetamine single-photon emission computed tomography
CEA: Carcinoembryonic antigen
CRT: Chemoradiation therapy
CT: Computed tomography
DM: Distal margin
FDG-PET: Fluorodeoxyglucose-positron emission tomography
HER2: Human epidermal growth factor receptor 2
IMRT: Intensity-modulated radiotherapy
INF: Pattern of infiltrating growth
MRI: Magnetic resonance imaging
PM: Proximal margin
por2: Non solid type poorly differentiated adenocarcinoma
PTX: Paclitaxel
sci: Scirrhous type
sig: Signet-ring cell carcinoma
T1b: Tumor invades the submucosal layer
T4a: Tumor exposes on the surface of serosa
TS-1^®^: Tegafur, Gimeracil, Oteracil potassium
